# Characteristics and Outcomes Associated with Cesarean Birth as Compared to Vaginal Birth at Mizan-Tepi University Teaching Hospital, Ethiopia

**DOI:** 10.26502/fjwhd.2644-28840060

**Published:** 2021-04-14

**Authors:** Margo S Harrison, Ephrem Kirub, Tewodros Liyew, Biruk Teshome, Andrea Jimenez-Zambrano, Margaret Muldrow, Teklemariam Yarinbab

**Affiliations:** 1 University of Colorado School of Medicine, Aurora, Colorado, USA; 2 Mizan-Tepi University, Department of Public Health, Mizan-Aman, Ethiopia; 3 Village Health Partnership, Denver, Colorado, USA

**Keywords:** Mode of Delivery, Cesarean Birth, Pregnancy Outcomes, Mizan-Aman, Ethiopia

## Abstract

**Introduction:**

The objective of this study was to observe characteristics and outcomes associated with cesarean birth as compared to vaginal birth.

**Methods:**

This study was a prospective hospital-based cross-sectional analysis of a convenience sample of 1, 000 women. Data was collected on admission, delivery, and discharge by trained physician data collectors on paper forms through chart review and patient interview.

**Results:**

Data on mode of delivery was available for 993/1000 women (0.7% missing data), 23.4% of whom underwent cesarean. These women were less likely to have labored (84.5% versus 87.4%), more likely to have been transferred (62.0% versus 45.2%), more likely to have been admitted in early labor (53.0% versus 48.6%), more likely to be in labor for longer than 24 hours (10.7% versus 3.3%) and were less likely to have multiple gestation (7.7% versus 3.9%), p < 0.05. In a Poisson model, history of cesarean (aRR 2.0, p < 0.001), transfer during labor (RR 1.5, p = 0.003), labor longer than 24 hours and larger birthweight (RR 2.7, p 0.001) were associated with an increased risk of cesarean.

**Conclusion:**

Our analysis suggests cesarean birth is being used among women with a history of prior cesarean and in cases of labor complications (prolonged labor or transfer), but fresh stillbirth is still common in this setting.

## 1. Introduction

Understanding and optimizing the use of cesarean birth is a global health priority and of great interest to the World Health Organization (WHO) [1, 2]. Globally, cesarean birth rates are increasing, although they remain below the recommended 10% rate in many sub-Saharan African settings [3, 4]. However, in many sub-Saharan African countries (and in other low- and middle-income countries) significant disparities in the use of cesarean birth exist [5, 6]. In many rural areas it is under-accessed and underused while in urban areas among certain populations it may be over-accessed and overused [5, 6]. Though cesarean birth can be essential to saving lives, it is also a major abdominal surgery with added risks above baseline pregnancy-related morbidity and mortality; as such, its use should be limited to medically indicated situations where optimization leads to a balance of risks and benefits [2, 5]. We wanted to observe the use of cesarean birth as compared to vaginal birth in a convenience sample of women delivering at a tertiary care facility in Mizan Aman, Mizan-Tepi University Teaching Hospital (MTUTH), in the Southern Nations, Nationalities, and People’s Region of Ethiopia (SNNPR). The objective of this analysis was to document mode of delivery at the hospital and characteristics and maternal and perinatal outcomes associated with cesarean birth. This was not hypothesis-driven research intended to fill a generalizable gap in knowledge, but rather an initial analysis intended to provide preliminary descriptive data that we can then use to study cesarean birth in more detail, prospectively.

Nonetheless, we did expect that the cesarean birth rate would be higher than that of the overall SNNPR region as MTUTH is a referral facility and that women with complications of pregnancy would give birth by cesarean. We also expected to observe that, similar to use of cesarean birth in other sub-Saharan African cohorts, cesarean may result in adverse pregnancy outcomes in women who necessitate the service [7, 8].

## 2. Methodology

### 2.1 Study design/setting

We conducted a prospective, hospital-based cross-sectional study at Mizan-Tepi University Teaching Hospital (MTUTH), which is located in Mizan-Aman in the Southern Nations, Nationalities, and People’s Region (SNNPR), Ethiopia.

### 2.2 Participants

The population for this study was a convenience sample of all pregnant women who consecutively delivered on labor and delivery at MTUTH between May 6 and October 21, 2019, which was the point at which 1, 000 women were included in the dataset. Only mothers who delivered after 28 completed weeks of pregnancy were included. Women were offered enrollment at admission and consented for de-identified collection of data regarding their delivery experience. Those who did not consent to have their data collected were not followed, accordingly.

### 2.3 Variables

We modeled our data collection documents after those used in the Global Survey for Maternal and Perinatal Health conducted by the World Health Organization, and those of the Global Network for Women’s and Children’s Health Research. Admission data included sociodemographic and early labor details, delivery data included details on the labor course and delivery mode, and the discharge form collected data on the postpartum course including maternal and perinatal complications and interventions. The forms used for data collection have been provided in an appendix. Common definitions for variables were defined by study authors prior to data collected, and a clear definition was included with the codebook.

### 2.4 Data sources/measurement

De-identified data was collected by highly trained physician data collectors with the intent of planning future quality improvement and research interventions. A combination of chart review and structured interview was used to collect information upon admission, delivery, and discharge. Data was collected on paper forms and the data collectors reviewed each other’s forms for completeness prior to data entry into REDCap for transmission and secure storage on a password protected server at the University of Colorado, Aurora, Colorado, USA [9]. Data from the paper forms were entered into REDCap 9.1.9, and STATA software version 15.2 (StataCorp LP, College Station, TX, USA) was used for analysis. This two-part data collection technique was used per preference of the data collectors who did not want to use personal cellular data to enter information.

### 2.5 Bias

We deliberately chose a cross-sectional, convenience, consecutive sample of patients for a baseline assessment of patients and their experience at MTUTH, which would have been biased by the sample that women who desired to deliver in a facility or were transferred there. No efforts were made to address any potential source of bias, but we did compare our population to the Ethiopian Demographic and Health Surveys in another analysis (under review) to describe how our sample may have differed.

### 2.6 Study size

MTUTH is a high-volume hospital that allowed for a large sample to be obtained over a relatively short amount of time. As a research team we agreed that collecting data on 1000 consecutive women over a number months women would hopefully give a less biased sample than a smaller sample collected over a shorter timeframe, but no formal sample size calculation was performed.

### 2.7 Statistical methods

As our objective was to observe characteristics and outcomes associated with cesarean birth compared to vaginal birth at MTUTH, we compared these two groups, specifically. Bivariate comparisons of sociodemographic, obstetric, labor, delivery, and pregnancy outcomes of women experiencing vaginal versus cesarean birth were performed, utilizing Fisher’s exact, Chi-squared, and Mann-Whitney U tests depending on the variables. For small cell size categorical variables the Fisher’s exact test was used, for categorical variables with larger counts and percentages, the chi-squared test was used, and more continuous variables the Mann-Whitney U test was employed. All covariates significant to p < 0.05 in bivariate comparisons were included in a multivariable Poisson model with robust error variance (because cesarean birth was prevalent) to determine which covariates were independently associated with cesarean birth. Subsequently, individual poisson regressions of maternal and perinatal outcomes (significant in the bivariate comparisons) were run with the outcomes as the dependent variable and cesarean birth as the independent variable, adjusted for all covariates significant in the original multivariable Poisson model, to describe the association between cesarean birth and adverse pregnancy outcomes.

### 2.8 Ethics

This quality improvement survey was given an exempt from human subjects’ research approval (COMIRB # 18–2738) by the University of Colorado and approval. Despite the quality improvement nature of the work and the fact that only de-identified data was collected, oral consent was obtained from each woman before any of her data was recorded.

## 3. Results

### 3.1 Participants

**Figure 1:**
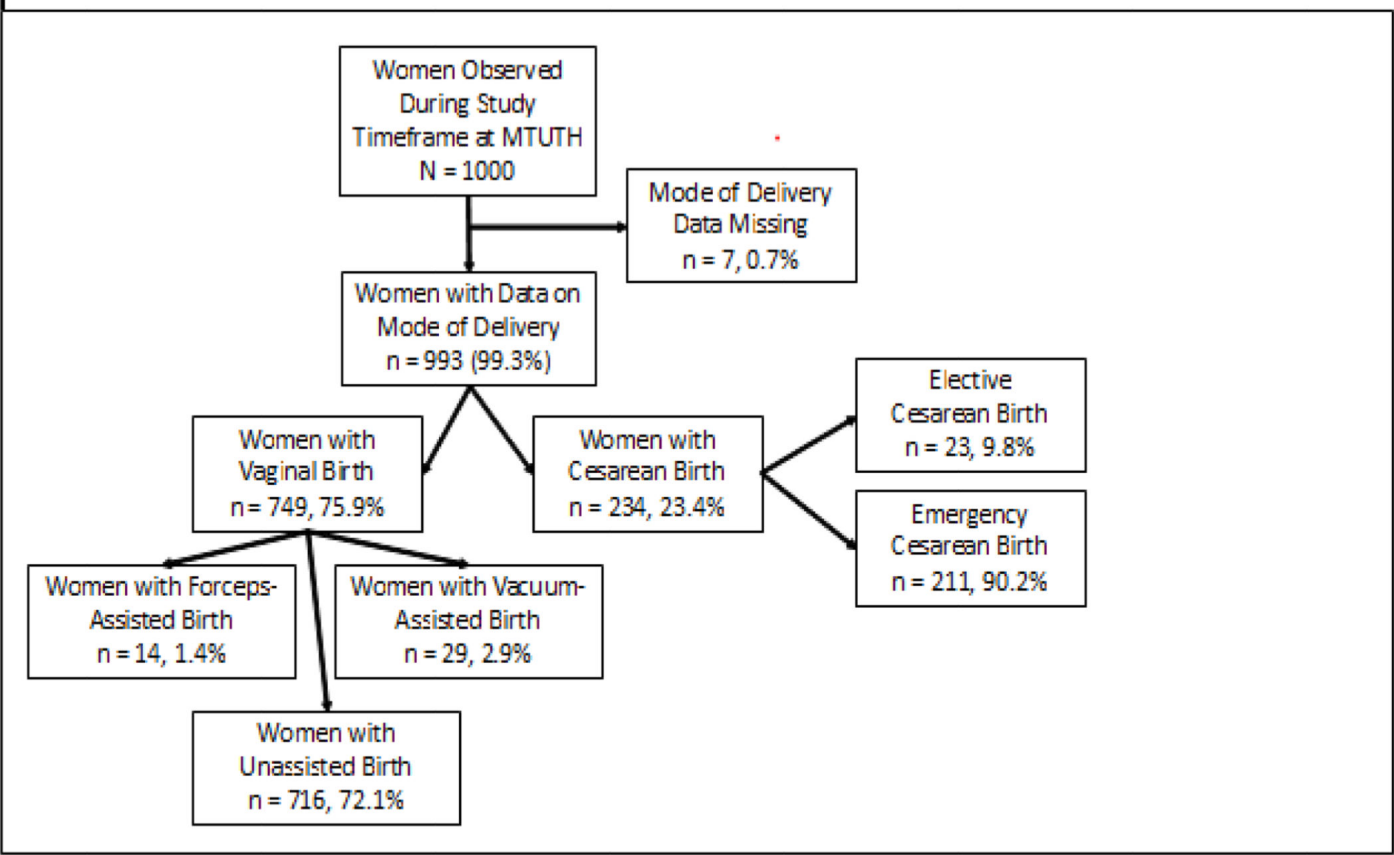
Study Population by Mode of Delivery (Cesarean versus Vaginal Birth).

As shown in Figure 1, 1, 000 women on whom data was collected, 993 (99.3% of the study population) included information on mode of delivery (how the woman gave birth).

### 3.2 Descriptive data

Almost a quarter (23.4%, n = 234) of women delivered by way of cesarean, with the remainder experiencing vaginal birth. The majority of women (90.2%) underwent cesarean birth in an emergent setting with the remaining (9.8%) of cesareans classified as elective. In terms of characteristics of the populations (Table 1), the median age of women delivering by cesarean was 25 (interquartile range [IQR] 21, 28), was almost statistically different than women delivering vaginally (p = 0.06). Compared to women who experienced vaginal birth, those that gave birth by cesarean were no different in education level, religion, relationship status, months since last delivery, gestational age, HIV status, or number of prenatal visits, p > 0.05. However, the proportion of cesarean birth was higher among women living in urban areas (65.0% versus 51.3%) and among women with a history of cesarean birth (14.1% versus 2.1%), p < 0.001. Outcome Data, Characteristics Associated with Cesarean Birth in Bivariate Comparisons (Table 2): Women who underwent cesarean birth compared to vaginal had a very different labor experience. Those experiencing cesarean birth were more likely to have been augmented (12.3% versus 12.1%) and less likely to be in spontaneous labor (84.5% versus 87.4%), more likely to have been transferred during labor (62.0% versus 45.2%), to be in latent versus active labor on admission as determined by cervical dilation (median of 3cm versus median of 4cm), to have a longer duration of labor (10.7% experience labor > 24 hours compared to 3.3%), and not have had their partograph completely utilized (30.8% versus 71.7%), p < 0.05. They also had less vaginal exams (median 2 exams versus 3 exams), experienced more antepartum hemorrhage (4.3% versus 1.6%), had larger babies (median 3200 grams versus 3000 grams), and were less likely to be carrying multiple gestations (7.7% not multiple gestation versus 3.9%), p < 0.05. Anesthesia use and delivery provider varied by vaginal versus cesarean birth, as expected.

Outcome Data, Outcomes Associated with Cesarean Birth in Bivariate Comparisons (Table 3): Complications of delivery also varied by mode of birth. Postpartum blood transfusion (4.7% versus 0.5%), antibiotic administration (30.3% versus 2.2%), and hypertensive treatment (2.6% versus 2.4%) were more common after cesarean birth, p < 0.05. Infants born to mothers by cesarean had a lower median 5-minute apgar score (8 versus 9) and were more likely to have a fresh stillbirth (6.0% versus 2.0%), p < 0.05. However, macerated stillbirths were more likely to be delivered vaginally (1.5% vaginally versus 0.4% by cesarean). Both maternal and neonatal length of hospitalization statistically significantly varied by mode of delivery, as expected. Outcome Data, Characteristics Associated with Cesarean Birth in Multivariable Model (Table 4A): Our final table illustrates the findings of both our Poisson model of characteristics associated with experiencing cesarean birth (4A) followed by independently run Poisson regressions where cesarean birth was the dependent variable testing its adjusted association with various maternal and perinatal outcomes (4B). All statistically significant variables with p < 0.05 in Tables 1 & 2 were included in the Poisson model with the addition of often important covariates age and parity, as both had borderline significance of p = 0.06. Only statistically significant associations are shared in Table 2A; characteristics associated with an increased risk of cesarean birth include: history of cesarean birth (aRR 2.0), having been transferred in labor (aRR 1.5), being in labor between 12 and 24 hours (aRR 1.3; compared to less than 12 hours) or being in labor longer than 24 hours (aRR 2.7; also compared to less than 12 hours), and carrying a fetus with a birthweight of ≥ 2500 grams (aRR 2.7; as compared to a baby with a birthweight < 2500 grams), although this last covariate is unknown until after birth, p < 0.05. Regarding characteristics that were independently associated with a reduced risk of cesarean birth, with each increasing centimeter of dilation of the cervix on admission to the hospital, there was an associated aRR of 0.9, and partograph completion had an aRR 0.6, p < 0.05 for both variables. Outcome Data, Outcomes Associated with Cesarean Birth in Individual, Adjusted Multivariable Models (Table 4B): Independent Poisson regressions of outcomes noted to be statistically significant in bivariate comparisons (Table 3) were run with cesarean birth as the dependent variable, adjusted for the statistically significant covariates (reported in Table 4A). Maternal interventions tested included postpartum blood transfusion, antibiotics, and hypertensive or anticonvulsant therapy. Cesarean birth was only associated with increased adjusted odds of antibiotic administration (aRR 10.5, p < 0.001), which could have been due to prophylactic administration [10]. The neonatal outcomes we tested were Apgar score, odds of live birth, and odds of the neonate being alive on discharge from the hospital; cesarean birth was associated with a reduced risk of having an Apgar score of greater than 7 at five minutes (aRR 0.9, p < 0.001).

## 4. Discussion

In summary, in our cross-sectional analysis of a convenience, consecutive sample of 1000 women who gave birth at MTUTH, we found that cesarean birth was prevalent (23.4%) in the 993 women with data on mode of birth, was more common in labors with complications (transfer, bleeding, prolongation), and in settings where it was used, maternal (bleeding, infection) and neonatal (lower 5-minute Apgar, fresh stillbirth) complications were more prevalent. Notable findings included the higher rate of cesarean birth among women from urban areas and the high prevalence of fresh stillbirths (compared to macerated) among cesarean as compared to vaginal births. Regarding the finding of a higher prevalence of cesarean birth among women living in urban areas, which for the purposes of this analysis represents the Mizan-Aman town from which about 45.0% of the population hails (data not shown), this is a common finding in the literature [11, 12]. Further analysis is needed comparing urban versus rural women undergoing cesarean birth in terms of pregnancy outcomes. If the rate of cesarean among urban women is higher without a resultant improvement in adverse outcomes such as stillbirth, the higher rate represents an unclear benefit. Conversely, if women in rural areas are experiencing more adverse outcomes with a lower cesarean birth rate, this would represent an unmet need for cesarean where a higher cesarean birth rate in this subgroup might contribute to improved outcomes. Though the overall rate of HIV positivity is low in the cohort (although higher than overall national estimates), it is notable that the mode of birth does not vary by HIV status [13]. More investigation into whether this is because pregnant women living with HIV are well-controlled with a low viral load that does not prohibit vaginal birth, or if it is because women were managed according to their obstetric indications for cesarean birth and not their HIV status, is warranted. This might be an area to pursue additional qualitative or survey research to understand how pregnant women living with HIV are managed in this setting.

Regarding obstetrical complications associated with cesarean birth, it is consistent with international literature that women with a history of cesarean birth, those transferred during labor, women admitted in early labor, and those with a prolonged labor course were more likely to experience cesarean birth [14–20]. What is less clear is the result suggesting that completion of the partograph (compared to non-use), reduced the risk of cesarean birth. We wonder if this is a spurious association, which might represent the fact that for women undergoing cesarean birth, their partograph was not used because they either had a truncated or nonexistent labor course. However, we feel it safe to recommend complete and high-quality use of the partograph given recent literature showing it reduces the rate of stillbirth (an adverse outcome common in this facility) [21]. It is well-known that women requiring cesarean birth experience more adverse outcomes than women undergoing vaginal birth [2, 5, 7, 8, 18, 22–24]. Cesarean birth is a major abdominal surgery that can result in adverse outcomes in itself, but it does follow logically that women with prolonged labor courses that required transfer who may have larger babies are more likely to experience labor dystocia, which might contribute to increased adverse outcomes. However, the finding that cesarean birth was associated with an increased risk of antibiotic use and not hemorrhage, for example, may support the known literature that endometritis is more common after cesarean than vaginal birth [25]. It is a limitation of this analysis that we did not define and observe sepsis rates, as in some settings it is standard practice for administration of postpartum prophylactic antibiotics so we do not know if this finding represents true infection [10]. A 6.0% rate of fresh stillbirth is concerning, but given our study was not designed to look at this outcome specifically, it is hard to draw and conclusions regarding the outcome. However, we would consider fresh stillbirth a modifiable risk factor. Intermittent fetal auscultation is a low-resource, low-complexity technology that the WHO has recommend during labor to monitor the fetal status [26, 27]. In light of the high stillbirth rate, this WHO recommendation may be indicated to ensure cesarean birth is used as a fetal life-saving measure when medically indicated [21]. MTUTH could consider a qualitative intervention to improve monitoring of laboring women or designing a prospective study to determine the root causes of stillbirth and any role under-monitoring might play.

This study is limited by the convenience, consecutive sampling technique and the variables selected for inclusion. The study sample may have been biased towards a population of women desiring to deliver in a facility or with high health-seeking behavior. The variables included those the study authors felt were relevant to labor and delivery care based on the literature and their own experience but may have neglected to include some other practitioners in the field consider to be relevant. For example, body mass index was not included, which would have been an informative variable, and given our finding about postpartum antibiotics, it would have been important to know whether they were administered prophylactically or for a confirmed diagnosis of postpartum endometritis. Strengths of the study include the large sample size and high-quality data collection by physicians with an intimate understanding of labor and delivery care.

## 5. Conclusion

In summary, cesarean birth appears to be being used at MTUTH in cases of labor complications (prolonged labor or transfer), which are medical indications. In this cohort, according to our analytic methods, there were minimal complications attributable to the procedure itself suggesting that high-quality cesareans are being performed at MTUTH. More research on the prevalence of stillbirth in this facility is warranted.

## Figures and Tables

**Table 1:** Sociodemographic and Obstetric Characteristics of Women Overall and by Mode of Delivery.

Characteristic	Overall Population	Bivariate Comparisons		
	N (%) N = 993	Vaginal Birth (n = 759, 75.9%)	Cesarean Birth (n = 234, 23.4%)	P-Value
Age in years, Median (IQR)	24 [20, 28]	24 [20, 28]	25 [21, 28]	0.06^a^
Missing	1 (0.1%)	*	*	
**Education**				0.60^b^
Unable to read & write	232 (23.4%)	173 (22.8%)	59 (25.2%)	
Read & write only	54 (5.4%)	46 (6.0%)	8 (3.4%)	
Primary school	396 (39.9%)	302 (39.8%)	94 (40.2%)	
Secondary school	138 (13.9%)	106 (14.0%)	32 (13.7%)	
Higher education	172 (17.3%)	131 (17.3%)	41 (17.5%)	
Missing	1 (0.1%)	1 (0.1%)	(0.0%)	
**Religion**				0.47^c^
Muslim	110 (11.1%)	79 (10.4%)	31 (13.3%)	
Orthodox Christian or Jehovah’s Witness	337 (33.9%)	258 (34.0%)	79 (33.8%)	
Protestant	545 (54.9%)	421 (55.5%)	124 (53.0%)	
Missing	1 (0.1%)	1 (0.1%)	0 (0.0%)	
**Relationship Status**				0.48^b^
Single	26 (2.6%)	22 (2.9%)	4 (1.7%)	
Not single	959 (96.6%)	730 (96.2%)	229 (97.9%)	
Missing	8 (0.8%)	7 (0.9%)	1 (0.4%)	
**Woreda**				<0.00 1^c^
Urban	541 (54.5%)	389 (51.3%)	152 (65.0%)	
Rural	452 (45.5%)	370 (48.7%)	82 (35.0%)	
**Parity**				0.06^c^
0	428 (42.8%)	325 (42.8%)	101 (43.3%)	
1	263 (26.3%)	199 (26.2%)	63 (26.9%)	
2	144 (14.4%)	120 (15.8%)	23 (9.8%)	
3	68 (6.8%)	50 (6.7%)	17 (7.3%)	
4	37 (3.7%)	29 (3.8%)	8 (3.4%)	
5+	58 (5.8%)	35 (4.6%)	22 (9.4%)	
Missing	2 (0.2%)	1 (0.1%)	0 (0.0%)	
**Months Since Last Delivery (parity 1+ n = 572)**				0.53^c^ 0.46^a^
< 24 months	46 (4.6%)	37 (4.9%)	9 (3.9%)	
24+ months	521 (52.5%)	398 (52.4%)	123 (52.6%)	
Missing	429 (42.9%)	324 (42.7%)	102 (43.6%)	
Median (IQR)	56 [36, 84]	60 [36, 84]	48 [36, 84]	
**Gestational Age Determination**				0.32^c^
Clinical Exam (Fundal Height)	472 (47.4%)	367 (48.6%)	102 (43.6%)	
Last Menstrual Period	66 (6.6%)	47 (6.2%)	19 (8.1%)	
Ultrasound	457 (45.9%)	341 (45.2%)	113 (48.3%)	
Missing	5 (0.5%)	4 (0.5%)	0 (0.0%)	
**History of Cesarean Birth**				<0.00 1^b^
0	942 (94.9%)	741 (97.6%)	201 (85.9%)	
1	44 (4.4%)	15(2.0%)	29 (12.4%)	
2+	5 (0.5%)	1 (0.1%)	4 (1.7%)	
Missing	2 (0.2%)	2 (0.3%)	0 (0.0%)	
**HIV+**				0.34^b^
Yes	21 (2.1%)	19 (2.5%)	2 (0.9%)	
No	964 (97.1%)	734 (96.7%)	230 (98.3%)	
Missing	8 (0.8%)	6 (0.8%)	2 (0.9%)	
**Number of Prenatal Visits**				0.38^c^
0	19 (1.9%)	11 (1.5%)	8 (3.4%)	
< 8	914 (92.0%)	703 (92.6%)	211 (90.2%)	
8+	55 (5.5%)	40 (5.3%)	15 (6.4%)	
Missing	5 (0.5%)	5 (0.7%)	0 (0.0%)	

a : Mann Whitney U test

b : Fisher’s Exact test

c : Chi-squared test

* unknown mode of delivery for missing data

Note: missing data was not included in bivariate comparisons and chi-squared testing was used for cell sizes above 5

**Table 2:** Antepartum, Labor, and Delivery Characteristics of Women by Mode of Delivery.

Characteristic	Overall Population	Bivariate Comparisons		
	N (%) N = 993	Vaginal Birth (n = 759, 75.9%)	Cesarean Birth (n = 234, 23.4%)	P-Value
**Onset of Labor**				<0.001^a^
Spontaneous	841 (84.7%)	663 (87.4%)	178 (84.5%)	
Augmented/Induced	123 (12.4%)	92 (12.1%)	31 (12.3%)	
Not Applicable (not in labor)	28 (2.8%)	3 (0.4%)	25 (2.8%)	
Missing	1 (0.1%)	1 (0.1%)	0 (0.0%)	
**Transferred During Labor**				<0.001^b^
No	505 (50.9%)	416 (54.8%)	89 (38.0%)	
Yes	488 (49.1%)	343 (45.2%)	145 (62.0%)	
**If Transferred, Transferring Facility (Transferred n = 481)**				1.0^a^
Health Center	471 (96.8%)	331 (97.9%)	140(97.9%)	
Private Clinic	7 (1.4%)	5 (1.5%)	2 (1.4%)	
Primary Hospital	3 (0.6%)	2 (0.6%)	1 (0.7%)	
**Cervical Exam on Admission**				<0.001^b^
<4 cm (latent labor)	493 (49.7%)	369 (48.6%)	124 (53.0%)	
4+ cm (active labor)	469 (47.2%)	376 (49.5%)	93 (39.7%)	
Missing or “Not done” or “Not Applicable”	31 (3.1%)	14 (1.8%)	17 (7.3%)	
**Fetal Heart Rate Auscultated at Admission**				0.26^a^
Yes	943 (95.0%)	724 (95.4%)	219 (93.6%)	
No (Fetal Demise)	39 (3.9%)	29 (3.8%)	10 (4.3%)	
Not Auscultated	2 (0.2%)	1 (0.1%)	1 (0.4%)	
Missing	9 (0.9%)	5 (0.7%)	4 (1.7%)	
**Duration of Labor**				<0.001^b^
Not Applicable (not in labor)	33 (3.3%)	6 (0.8%)	27 (11.5%)	
< 12 hours	510 (51.2%)	424 (55.9%)	86 (36.8%)	
12 – 24 hours	400 (40.2%)	304 (40.1%)	96 (41.0%)	
24+ hours	50 (5.2%)	25 (3.3%)	25 (10.7%)	
**Partograph Used**				<0.001^a^
Not Applicable (not in labor)	271 (27.3%)	133 (17.5%)	138 (56.0%)	
No	94 (9.5%)	74 (9.8%)	20 (8.6%)	
Yes, Incomplete	10 (1.0%)	6 (0.8%)	4 (1.7%)	
Yes, Complete	616 (62.0%)	544 (71.7%)	72 (30.8%)	
Missing	2 (0.2%)	2 (0.3%)	0 (0.0%)	
Number of Vaginal Exams Median (IQR)	3 [2, 3]	3 [2, 4]	2 [1, 3]	<0.001^c^
Missing	5 (0.5%)	1 (0.1%)	4 (1.7%)	
**Antepartum Hemorrhage**				0.04^b^
No	970 (97.7%)	746 (98.3%)	224 (95.7%)	
Yes	22 (2.2%)	12 (1.6%)	10 (4.3%)	
Missing	1 (0.1%)	1 (0.1%)	0 (0.0%)	
**Chorioamnionitis**				0.63^a^
No	987 (0.6%)	755 (99.5%)	987 (99.2%)	
Yes	6 (99.3%)	4 (0.5%)	6 (0.9%)	
**Antepartum Pre-eclampsia/Eclampsia/Chronic Hypertension**				0.68^b^
No	945 (95.2%)	724 (95.4%)	221 (94.4%)	
Yes	47 (4.7%)	34 (4.5%)	13 (5.6%)	
Missing	1 (0.1%)	1 (0.1%)	0 (0.0%)	
**Anesthesia for Birth**				<0.001^a^
None	672 (67.7%)	671 (88.4%)	1 (0.4%)	
Any	321 (32.4%)	88 (11.6%)	233 (99.6%)	
**Provider who Delivered the Infant**				<0.001^a^
Midwife	730 (73.2%)	729 (96.1%)	1 (0.4%)	
General Practitioner	10 (1.0%)	9 (1.2%)	1 (0.4%)	
Integrated Emergency and Surgical Officer	242 (24.3%)	20 (2.6%)	222 (95.3%)	
Ob/Gyn Resident or Attending	10 (1.0%)	1 (0.1%)	9 (3.9%)	
Missing	1 (0.1%)	*	*	
**Gestational Age at Delivery**				0.29^b^
Preterm (< 37 weeks)	103 (10.4%)	85 (11.2%)	18 (7.7%)	
Term (≥ 37 weeks)	887 (89.2%)	672 (88.5%)	215 (91.9%)	
Missing	3 (0.3%)	2 (0.3%)	1 (0.4%)	
**Birthweight (grams)**				0.03^b^
<2500	70 (7.1%)	57 (7.5%)	13 (5.6%)	
≥ 2500	873 (87.9%)	671 (88.4%)	202 (86.3%)	
Missing	50 (5.0%)	31 (4.1%)	19 (8.1%)	
**Neonatal Sex**				0.20^b^
Male	530 (53.4%)	401 (55.1%)	129 (60.0%)	
Female	413 (41.6%)	327 (44.9%)	86 (40.0%)	
Missing	50 (5.0%)	*	*	
**Multiple Gestation**				0.02^b^
Yes	945 (95.2%)	729 (96.1%)	216 (92.3%)	
No	48 (4.8%)	30 (3.9%)	18 (7.7%)	
Missing	0 (0.0%)	0 (0.0%)	0 (0.0%)	

a : Fisher’s Exact test

b : Chi-squared test

c : Mann-Whitney U test

* unknown mode of delivery for missing data

Notes: Categories for duration of labor were chosen by study team and not per a reference article; we suspect the 1 woman who reportedly underwent cesarean birth without anesthesia is likely an erroneous data point; an Integrated Emergency Surgical Officer is a non-physician surgeon in the Ethiopian surgical workforce; missing data not included in bivariate comparisons and chi-squared testing was used for cell sizes above 5.

**Table 3:** Postpartum Interventions and/or Complications of Women and Infants by Mode of Delivery.

Characteristic	Overall Population	Bivariate Comparisons			
	N (%) N = 993	Vaginal Birth (n = 759, 75.9%)	Cesarean Birth (n = 234, 23.4%)		P-Value
**Postpartum Maternal Interventions and/or Complications**					
**Postpartum Blood Transfusion**					<0.001^a^
No	976 (98.3%)	755 (99.5%)	221 (94.4%)		
Yes	15 (1.5%)	4 (0.5%)	11 (4.7%)		
Missing	2 (0.2%)	0 (0.0%)	2 (0.9%)		
**Postpartum Antibiotics**					<0.001^b^
No	900 (90.6%)	740 (97.5%)	160 (68.4%)		
Yes	88 (8.9%)	17 (2.2%)	71 (30.3%)		
Missing	5 (0.5%)	2 (0.3%)	3 (1.3%)		
**Postpartum Hypertensive Treatment**					0.04^b^
No	967 (97.4%)	741 (97.6%)	226 (96.6%)		
Yes	24 (2.4%)	18 (2.4%)	6 (2.6%)		
Missing	2 (0.2%)	0 (0.0%)	2 (0.9%)		
**Postpartum Anticonvulsant Treatment**					0.05^a^
No	965 (97.2%)	737 (97.1%)	228 (97.4%)		
Yes	26 (2.6%)	22 (2.9%)	4 (1.7%)		
Missing	2 (0.2%)	0 (0.0%)	2 (0.9%)		
**Maternal Length of Hospitalization Median (IQR)**	1 [1, 3]	1 [1, 1]	3 [3, 4]		<0.001^c^
Missing	0 (0.0%)	0 (0.0%)	0 (0.0%)		
**Neonatal Interventions and/or Complications**					<0.001^c^
Five-Minute Apgar Score Median (IQR)	9 [8, 9]	9 [8, 9]	8 [7, 9]		
Missing	45 (4.5%)	30 (3.0%)	18 (1.8%)		
**Stillbirth**					0.001^a^
Yes, Fresh	29 (2.9%)	15 (2.0%)	14 (6.0%)		
Yes, Macerated	12 (1.2%)	11 (1.5%)	1 (0.4%)		
No	903 (90.9%)	702 (92.5%)	201 (85.9%)		
Missing	49 (4.9%)	31 (4.1%)	18 (7.7%)		
**Bag & Mask Resuscitation**					0.79^a^
No	977 (98.4%)	746 (98.3%)	231 (98.7%)		
Yes	12 (1.2%)	9 (1.2%)	3 (1.3%)		
Missing	4 (0.4%)	4 (0.5%)	0 (0.0%)		
**Intranasal Oxygen**					0.79^a^
No	970 (97.7%)	740 (97.5%)	230 (98.3%)		
Yes	20 (2.0%)	16 (2.1%)	4 (1.7%)		
Missing	3 (0.3%)	3 (0.4%)	0 (0.0%)		
**Continuous Positive Airway Pressure (CPAP)**					0.18^a^
No	980 (97.7%)	750 (98.8%)	230 (98.3%)		
Yes	9 (2.0%)	5 (0.7%)	4 (1.7%)		
Missing	4 (0.3%)	4 (0.5%)	0 (0.0%)		
**Intravenous Fluid Administration**					0.18^b^
No	959 (96.6%)	731 (96.3%)	228 (97.4%)		
Yes	31 (3.1%)	25 (3.3%)	6 (2.6%)		
Missing	3 (0.3%)	3 (0.4%)	0 (0.0%)		
**Antibiotics**					0.85^b^
No	952 (95.9%)	727 (95.8%)	225 (96.2%)		
Yes	34 (3.4%)	26 (3.4%)	8 (3.4%)		
Missing	7 (0.7%)	6 (0.8%)	1 (0.4%)		
**Blood Transfusion**					1.0^a^
No	982 (98.9%)	756 (98.8%)	232 (99.2%)		
Yes	4 (0.4%)	2 (0.4%)	1 (0.4%)		
Missing	7 (0.7%)	1 (0.8%)	1 (0.4%)		
**Neonate Status on Day of Discharge**					0.07^b^
Dead*	55 (5.5%)	35 (4.6%)		20 (8.6%)	
Alive	933 (94.0%)	720 (94.9%)		213 (91.0%)	
Missing	5 (0.5%)	4 (0.5%)		1 (0.4%)	
Neonatal Length of Hospitalization, Median (IQR)	1 [1, 3]	1 [1, 1]		3 [3, 4]	<0.001^c^
Missing	20 (2.0%)	13 (1.7%)		7 (3.0%)	

a : Fisher’s Exact test

b : Chi-squared test

c : Mann-Whitney U test

* Total number dead, including stillbirths

Note: missing data were not included in bivariate comparisons, and chi-squared testing was used for cell sizes above 5

**Table 4:** A) Multivariable Model of Characteristics Associated with Cesarean Birth and; B) How Outcomes are Impacted by Undergoing Cesarean Birth.

Characteristic	RR	CI	P-Value
(A) Multivariable Poisson Model with Robust Error Variance of Characteristics Associated with Cesarean Birth*			
History of Cesarean Birth	2.0	1.5, 2.7	<0.001
Transferred in Labor	1.5	1.1, 1.9	0.003
Increased Cervical Dilation on Admission	0.9	0.9, 0.9	<0.001
**Compared to Less Than 12 hours of Labor:**			
12 – 24 hours	1.3	1.1, 1.9	0.03
> 24 hours	2.7	1.8, 3.9	<0.001
**Compared to Non-Use of the Partograph:**			
Complete Use	0.6	0.4, 0.9	0.03
Birthweight ≥2500	2.7	1.5, 4.8	0.001
(B) Individual Multivariable Poisson Models with Robust Error Variance, Adjusted for Significant Findings in Table 4A, to Determine Association of Cesarean Birth with Outcomes Significant in Bivariate Comparisons (Table 3)			
Characteristic	RR	CI	P-Value
**Maternal Outcomes**			
Needing Maternal Blood Transfusion	3.2	0.7, 14.0	0.1
Needing Postpartum Antibiotics	10.5	6.1, 18.1	<0.001
Needing Postpartum Hypertensive Therapy	1.1	0.3, 3.3	0.9
Needing Postpartum Anticonvulsant Therapy	0.4	0.1, 1.7	0.2
**Neonatal Outcomes**			
Having a Higher Apgar Score	0.9	0.9, 0.98	0.01
Live Birth	1.0	0.9, 1.1	0.6
Being Alive at Discharge from the Hospital	1.0	0.9, 1.1	0.7

* variables included in the model without an association with the outcome: age, urban/rural residence, parity, onset of labor (spontaneous or not), incomplete partograph use, number of vaginal exams, antepartum hemorrhage, number of fetuses delivered

## Appendix

**Appendix:** Degrees of Freedom, Test Statistic, and Cramer’s V for Each Covariate

Characteristic	Degrees of Freedom	Test Statistic	Cramer’s V Test of Correlation with Mode of Birth
Age	-	Z = −1.9	0.1
Education	5	3.1	0.06
Religion	1	13.9	−0.1
Relationship Status	2	1.5	0.5
Woreda	2	1.6	0.04
Parity	6	12.1	0.06
Months Since Last Delivery (parity 1+ n = 572)	-	Z = 0.5	0.2
Gestational Age Determination	2	2.29	0.05
History of Cesarean Birth	2	55.2	0.2
HIV+	1	2.3	−0.04
Number of Prenatal Visits	-	Z = −0.9	0.2

a : Mann Whitney U test;

b : Fisher’s Exact test;

c : Chi-squared test

**Table:**

Characteristic	Degrees of Freedom	Test Statistic	Cramer’s V Test of Correlation with Mode of Birth
Onset of Labor	2	70.0	0.3
Transferred During Labor	1	20.3	0.1
If Transferred, Transferring Facility (Transferred n = 481)	2	0.02	0.007
Cervical Exam on Admission	-	Z = 2.73	0.2
Fetal Heart Rate Auscultated at Admission	2	0.9	0.03
Duration of Labor	3	94.3	0.3
Partograph Used	3	162.4	0.4
Number of Vaginal Exams	-	Z = 4.5	0.4
Antepartum Hemorrhage	1	6.0	0.08
Chorioamnionitis	1	0.3	0.02
Antepartum Pre-eclampsia/Eclampsia/Chronic Hypertension	1	0.5	0.02
Anesthesia for Birth	1	632.8	0.8
Delivery Provider	3	874.3	0.9
Gestational Age at Delivery	-	Z = −2.3	0.1
Birthweight (grams)	-	Z = −3.1	0.3
Neonatal Sex	1	1.6	−0.04
Multiple Gestation	1	5.4	0.07

a : Fisher’s Exact test;

b : Chi-squared test;

c : Mann-Whitney U test

**Table:**

Characteristic	Degrees of Freedom	Test Statistic	Cramer’s V Test of Correlation with Mode of Birth
Postpartum Blood Transfusion	1	1.2	−0.03
Postpartum Antibiotics	1	0.6	−0.02
Postpartum Hypertensive Treatment	1	0.03	0.006
Postpartum Anticonvulsant Treatment	1	1.0	−0.03
Maternal Length of Hospitalization	-	Z = −25.1	0.8
Five-Minute Apgar Score	-	Z = 8.7	0.4
Stillbirth	2	12.2	0.1
Bag & Mask Resuscitation	1	0.01	0.004
Intranasal Oxygen	1	0.1	−0.01
Continuous Positive Airway Pressure (CPAP)	1	2.2	0.05
Intravenous Fluid Administration	1	0.3	−0.01
Antibiotics	1	<0.001	< - 0.001
Blood Transfusion	1	0.004	0.002
Neonate Status on Day of Discharge	1	5.3	−0.07
Neonatal Length of Hospitalization	-	Z = −21.5	0.8

a : Fisher’s Exact test;

b : Chi-squared test;

c : Mann-Whitney U test
